# Preferential Ty1 retromobility in mother cells and nonquiescent stationary phase cells is associated with increased concentrations of total Gag or processed Gag and is inhibited by exposure to a high concentration of calcium

**DOI:** 10.18632/aging.101402

**Published:** 2018-03-21

**Authors:** Andrew C. Peifer, Patrick H. Maxwell

**Affiliations:** 1 Department of Biological Sciences, Rensselaer Polytechnic Institute, Troy, NY 12180, USA; 2 Wadsworth Center, Division of Genetics, Albany, NY 12208, USA

**Keywords:** replicative aging, quiescence, retrotransposon, *Saccharomyces cerevisiae*, Ty1

## Abstract

Retrotransposons are abundant mobile DNA elements in eukaryotic genomes that are more active with age in diverse species. Details of the regulation and consequences of retrotransposon activity during aging remain to be determined. Ty1 retromobility in *Saccharomyces cerevisiae* is more frequent in mother cells compared to daughter cells, and we found that Ty1 was more mobile in nonquiescent compared to quiescent subpopulations of stationary phase cells. This retromobility asymmetry was absent in mutant strains lacking *BRP1* that have reduced expression of the essential Pma1p plasma membrane proton pump, lacking the mRNA decay gene *LSM1*, and in cells exposed to a high concentration of calcium. Mother cells had higher levels of Ty1 Gag protein than daughters. The proportion of protease-processed Gag decreased as cells transitioned to stationary phase, processed Gag was the dominant form in nonquiescent cells, but was virtually absent from quiescent cells. Treatment with calcium reduced total Gag levels and the proportion of processed Gag, particularly in mother cells. We also found that Ty1 reduced the fitness of proliferating but not stationary phase cells. These findings may be relevant to understanding regulation and consequences of retrotransposons during aging in other organisms, due to conserved impacts and regulation of retrotransposons.

## Introduction

Retrotransposons copy their RNA transcripts through reverse transcription to produce cDNA molecules that are inserted into genomes, and they have been associated with aging in several model organisms [1]. Retrotransposon expression, integration of new cDNA copies (retrotransposition), or both are increased with age in *S. cerevisiae*, *C. elegans*, *D. melanogaster*, mice, and normal diploid human cells [2]. Correlations between retrotransposon activity and aging-related genomic instability have also been observed [3–6].

Changes in chromatin and siRNA-silencing pathways may contribute to the increased activity of retrotransposons with age [7], but specific impacts and regulation of retrotransposons during aging are not well understood.

*S. cerevisiae* Ty1 retromobility (incorporation of cDNA into the genome by integration or recombination) is elevated as stationary phase cells and dividing mother cells age [3,5]. Ty1 elements are retroviral-like retrotransposons that have long terminal repeats at each of their ends and two overlapping open reading frames that are translated into Gag and Gag-Pol fusion proteins [8]. Translation of Ty1 Gag occurs at the endoplasmic reticulum (ER) and Gag translocates through the ER before associating with Ty1 mRNA in Gag-RNA foci called retrosomes [9]. Retrosomes are precursors to virus-like particles (VLPs) that are formed by Gag and package Ty1 mRNA and Gag-Pol [8,10,11]. A protease domain in Pol processes the initial p49 form of Gag into a p45 form, cleaves Gag from Gag-Pol, and processes Pol into protease, integrase, and reverse transcriptase/RNase H domains during VLP maturation [8]. Reverse transcriptase/RNase H synthesizes a cDNA from Ty1 mRNA in VLPs that can then be integrated at a new genomic site by the integrase domain [8]. Ty1 also encodes a truncated version of Gag, p22, that is translated from an internally initiated mRNA and which has a dominant-negative influence in trans on Ty1 retromobility in a Ty1 copy-dependent manner [12]. Increased Ty1 retrotransposition frequency in aging mother cells relative to their daughter cells was not found to be due to substantial asymmetry in Ty1 mRNA or Gag accumulation in mother cells versus their daughter cells, but was correlated with a large increase in Ty1 cDNA in mothers compared to daughters [5].

It remains to be determined whether known asymmetries between yeast mother and daughter cells or a Ty1-specific mechanism is responsible for the asymmetry in retromobility between mothers and daughters. An early asymmetry during yeast replicative aging is the increase in cytoplasmic pH in mothers compared to daughters due to the accumulation of the plasma membrane proton transporter Pma1p in mothers [13]. This asymmetry contributes to decreased mitochondrial function and decreased vacuole acidity in mother cells [13]. Decreased vacuole acidity might decrease autophagy in mother cells, and Ty1 is inhibited by autophagy [14]. Diffusion barriers prevent certain components of mother cells’ cytoplasm from being transmitted to daughter cells, such as the ER diffusion barrier that prevents misfolded ER proteins from being inherited by daughters [15]. Protein aggregates are retained in mother cells during division through association with organelles, which depends on the function of the Hsp104p protein disaggregase [16]. It is not known whether factors contributing to these diffusion barriers can also restrict Ty1 retrosomes or VLPs to mother cells.

*S. cerevisiae* cells respond to depletion of nutrients during stationary phase by entering a quiescent state that is characterized by a temporary exit from the cell cycle until conditions become favorable for growth [17]. Quiescence entry in yeast is associated with highly asymmetric cell divisions [18]. Only a subpopulation of stationary phase cells undergoes appropriate adaptations to become quiescent, and only this subpopulation is referred to as quiescent (Q) cells, while all other stationary phase cells are referred to as nonquiescent (NQ) cells [19]. Yeast Q and NQ cells can be fractionated by density, and many mRNA molecules are found in a protein-bound state in Q cells but are in a protein-free state in NQ cells, including Ty1 mRNA [19,20]. Ty1 retromobility in Q and NQ cells was not previously investigated, though.

We report that mRNA decay factors, pH homeostasis, and calcium regulate asymmetry in Ty1 retromobility between mother and daughter cells and between NQ and Q cells. Exposure to high calcium reduced overall Ty1 Gag levels, but also increased the proportion of unprocessed Gag or a posttranslationally modified form of Gag originally observed in G1-arrested cells [21]. Mother cells had higher total Gag than daughter cells, and the proportion of processed p45-Gag decreased as cells entered stationary phase, becoming virtually undetectable in Q cells. The rate of Ty1 retromobility was much higher in exponential phase cells than early stationary phase cells, and Ty1 reduced the fitness of proliferating but not stationary phase cells. Similarities between retrotransposon regulation and asymmetries during cell division between yeast and mammalian cells make this work potentially relevant to investigating retrotransposition in asymmetrically dividing stem cells [22–24].

## RESULTS

### pH homeostasis regulates Ty1 retromobility asymmetry between mothers and daughters

**Figure 1:**
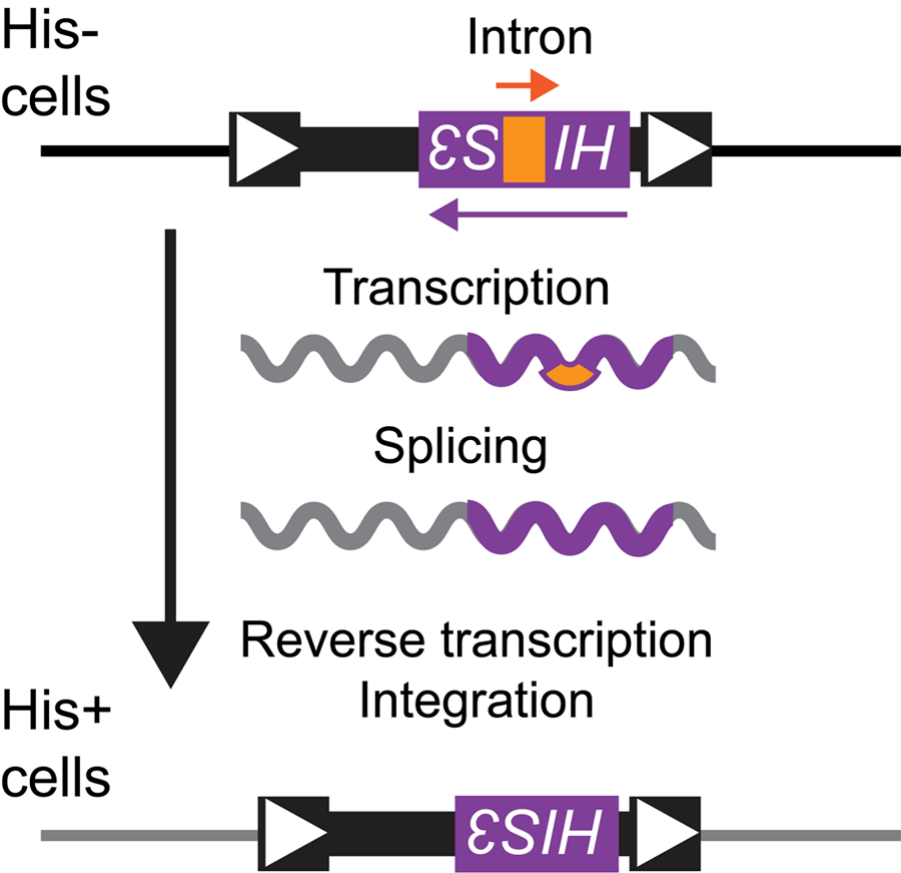
**Ty1*his3AI* assay for measuring Ty1 retromobility**. A strain with a deletion at the endogenous *HIS3* locus harbors a chromosomal Ty1 element with the *his3AI* indicator gene between Ty1 coding sequences and the 3′ long terminal repeat (white arrowheads indicate long terminal repeats). The strain cannot grow in the absence of histidine because the *HIS3* sequence within Ty1 is disrupted by an oppositely oriented intron (*AI*, labeled “Intron” in drawing) that is not spliced when transcription initiates from the *HIS3* promoter. Transcription of Ty1*his3AI* from the Ty1 promoter allows splicing of *AI*, and reverse transcription produces a Ty1*HIS3* cDNA that can be incorporated into the genome. Cells that acquire Ty1*HIS3* through a retromobility event express *HIS3* and gain a His^+^ prototroph phenotype.

**Figure 2:**
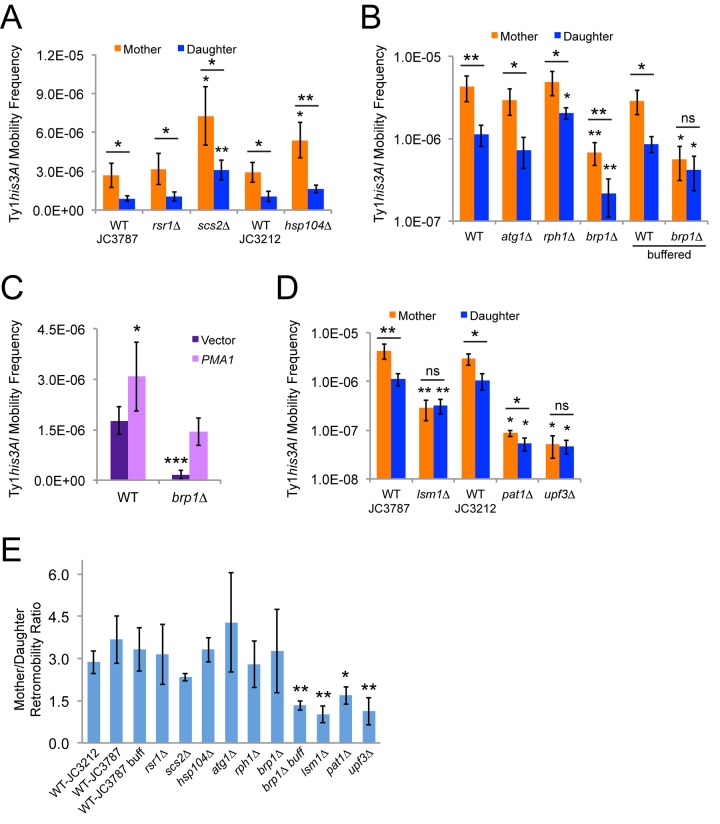
**Mother-daughter asymmetry in Ty1 retromobility depends on pH homeostasis and mRNA decay factors.** (**A-B**) His^+^ frequencies for mother and daughter cells of the indicated genotypes separated by magnetic cell sorting after growth in standard YPD medium or in YPD buffered to pH 7.1 with 20 mM sodium phosphate (buffered). Mutants are shown to the right of their corresponding wild type strain (JC3787 or JC3212 in panel **A**, JC3787 in panel **B**). Results from three to six trials per genotype are shown. (**C**) His^+^ frequencies for cells of the indicated genotypes grown to mid-exponential phase with a low copy vector or a low copy vector with a copy of *PMA1* under the control of its native promoter. (**D**) Same as for panels **A** and **B**. JC3787 and JC3212 wild type data are the same as for panels **B** and **A**, respectively. (**E**) Ratios of Ty1 retromobility frequencies in mothers divided by the frequencies in daughters for all the strains and conditions from panels **A**, **B**, and **D**. All graphs show mean values and standard deviations. Single, double, or triple asterisks indicate p<0.05, 0.01, or 0.001, respectively, and “ns” indicates p>0.05. Asterisks over individual columns indicate differences compared to the relevant control or wild type value. Horizontal bars indicate comparisons between mother and daughter cells for a given genotype or condition. Note that the y-axis in panels **B** and **D** is a log scale to show significant variation in frequencies.

Since Ty1 mRNA and Gag-GFP levels are similar in mother and daughter cells but cDNA levels are much higher in mother cells [5], we tested whether candidate genes contributing to asymmetries between mothers and daughters that could influence intermediate steps in the Ty1 retromobility cycle regulate Ty1 retromobility asymmetry. Two related *S. cerevisiae* strains that each harbor the same chromosomal Ty1*his3AI* element were used for these experiments, permitting Ty1 retromobility to be quantified based on the frequency with which cells become His^+^ prototrophs able to grow in the absence of histidine (Fig. 1) [25]. Initial cell populations were grown on solid medium at 30˚C to inhibit Ty1 retromobility [26], surface-labeled with biotin, and grown in liquid medium for approximately four population doublings at 20˚C to allow efficient Ty1 retrotransposition. Cells were then incubated with anti-biotin magnetic beads and passed through magnetic columns to separate biotin-labeled mother cells from their unlabeled daughter cells [5]. Staining of bud scars with a fluorescent conjugate of wheat germ agglutinin (WGA-Alexa 488) indicated that at least 80 ± 11% of eluted wild type cells and 67 ± 6.4% to 81 ± 5.2% of eluted mutant cells were mother cells approximately four generations old (see Methods), similar to previous results for mother cells in this age range [5]. Translation of Ty1 mRNA occurs at the ER and retrosomes (Ty1 Gag-RNA foci) have been proposed to form at the ER [9]. We therefore tested whether genes that contribute to the ER diffusion barrier between mothers and daughters, *RSR1* (*BUD1*) and *SCS2*, or to the retention of protein aggregates that are formed at the ER surface in mother cells, *HSP104*, contribute to preferential retrotromobility in mother cells [15,27,28]. The mother to daughter cell ratios for Ty1 retromobility frequency were very similar for *rsr1∆* (*bud1∆*), *scs2∆*, and *hsp104∆* mutants compared to the wild type strains (Figs. 2A and 2E), which does not support a role for these genes in retromobility asymmetry. The ER diffusion barrier and efficient retention of protein aggregates in mothers are therefore not needed for Ty1 retromobility asymmetry.

Early changes in mother cells during replicative aging include decreased vacuole acidity and increased cytoplasmic pH as a result of higher levels of the plasma membrane proton pump Pma1p in mother cells compared to daughter cells [13]. Pma1p is an essential protein, but deleting the *BRP1* dubious ORF that is upstream of *PMA1* reduces Pma1p protein level and activity and also reduces Ty1 retromobility [29,30]. The yeast vacuole is the site of degradation of cellular components through autophagy, and autophagy has been reported to inhibit Ty1 retromobility [14]. We found that *atg1∆* mutants deficient for autophagy and *rph1∆* mutants with elevated basal levels of autophagy still exhibited retromobility asymmetry between mother and daughter cells (Fig. 2B and 2E) [31,32]. There was a statistically significant increase in retromobility in *rph1∆* daughter cells, but it is not clear that such a modest change is biologically significant. Retromobility asymmetry was also still observed when *brp1∆* mutants defective for pH homeostasis were grown in standard rich medium (YPD), though overall Ty1 retromobility was strongly reduced and a log scale was used for the y-axis of this graph to facilitate seeing the asymmetry at these low frequencies (Fig. 2B). However, Ty1 retromobility asymmetry was no longer observed in *brp1∆* mutants when cells were grown in YPD medium buffered to pH 7.1 ± 0.14 (Figs. 2B and 2E), compared to an unbuffered initial pH of 5.9 ± 0.11. Loss of asymmetry resulted from retromobility not being as strongly inhibited in the *brp1∆* daughter cells with buffering, indicating that higher medium pH might compromise the ability of mutant daughter cells to maintain a low pH or another aspect of their intracellular environment that restricts Ty1. This change in pH did not significantly alter the overall level of Ty1 retromobility in these cells, though. We expressed *PMA1* from its native promoter on a low-copy plasmid in wild type and *brp1∆* mutant cells, which modestly increased retromobility in wild type cells and restored retromobility in *brp1∆* mutants to wild type levels (Fig. 2C). These results indicate that changes in Ty1 retromobility in *brp1∆* mutants result from decreased *PMA1* expression, and that pH homeostasis can regulate Ty1 retromobility asymmetry between mother and daughter cells.

### mRNA decay factors are required for Ty1 retromobility asymmetry between mothers and daughters

Ty1 retrosomes (Gag-RNA foci) are more prevalent in budding mother cells [10], and Ty1 cDNA levels are much higher in aging mothers compared to their daughters [5]. Core P-body factors and mRNA decay factors are needed for efficient Ty1 retrosome and cDNA formation, and absence of such factors substantially inhibits retromobility [10,11], so we tested whether they contribute to retromobility asymmetry. Overall retromobility was strongly reduced in *lsm1∆* and *upf3∆* mutants defective for deadenylation-dependent mRNA decay and nonsense-mediated mRNA decay, respectively [33,34], but mother cells were disproportionately affected which abolished retromobility asymmetry (Figs. 2D and 2E). Note that a log scale was used for the y-axis in Fig. 2D to more clearly observe the loss of asymmetry at the low frequencies observed in the mutants. Retromobility was also reduced in *pat1∆* mutants that do not express a core P-body protein that associates with the Lsm1-7p complex [33], with a greater relative inhibition in mother cells that significantly reduced the mother/daughter retromobility ratio (Figs. 2D and 2E). These data support a role for mRNA decay factors in the regulation of retromobility asymmetry.

### Exposure to a high concentration of calcium disproportionally inhibits Ty1 in mother cells to abolish retromobility asymmetry

Induction of P-bodies by glucose deprivation inhibits Ty1 retrosome formation [10], so we tested whether inducing P-bodies would prevent retromobility asymmetry. We exposed cells to glucose deprivation, high calcium chloride, or high sodium chloride levels, all of which induce P-bodies [35], to verify that P-body induction would reduce Ty1 retromobility. Retromobility frequencies were measured in exponential phase cells grown in YPD medium, then portions of those cell populations were grown for eight hours in either rich medium lacking glucose, YPD with 10 or 100 mM calcium chloride, or YPD with 0.5 M sodium chloride, and retromobility frequencies were measured again. Eight-hour incubations were used in order to allow cells to progress through the cell cycle, since Ty1 is inhibited in G1 [21], and because cells were previously grown for several hours at 20˚C to observe induction of Ty1 by acute radiation exposure [36], indicating that the retromobility cycle could require hours for completion. Relative retromobility frequencies were calculated by dividing the final frequencies by the initial frequencies to more easily compare the effects of each treatment.

**Figure 3:**
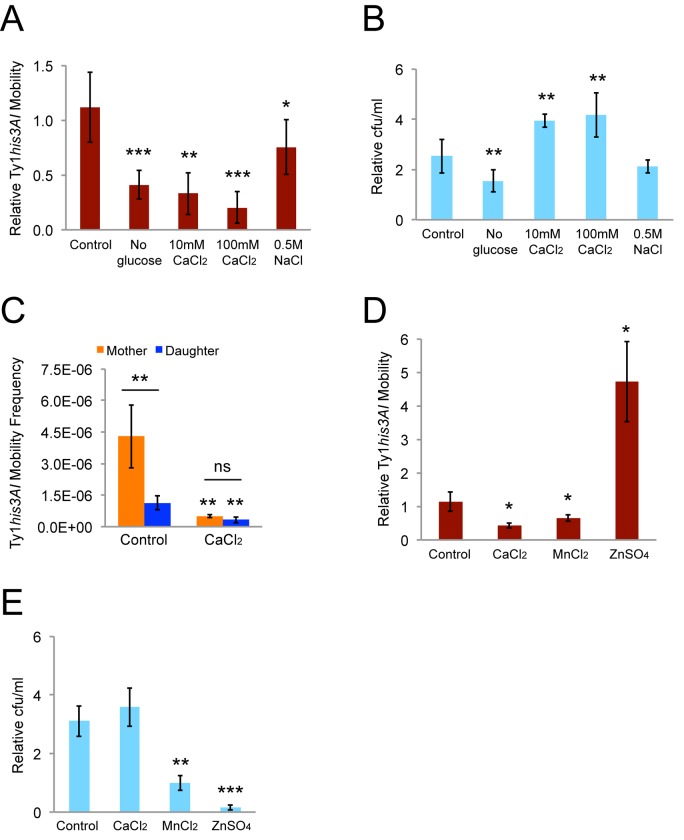
**Growth in medium with a high calcium concentration abolishes mother-daughter retromobility asymmetry.** (**A**) Relative His^+^ frequencies after eight-hour incubations in conditions known to induce P-bodies normalized to the frequencies immediately prior to the eight-hour incubations. Results are for 11 control trials and three to seven trials in the other conditions. (**B**) Colony forming units (cfu) per mL after the eight-hour incubations normalized to the cfu/mL immediately prior to the incubations for the trials shown in panel **A**. (**C**) His^+^ frequencies for sorted mother and daughter cells without (six trials) or with (four trials) chronic exposure to 100 mM calcium chloride. Control data are the same as from Fig. 2B. (**D**) Relative His^+^ frequencies after eight-hour incubations in 10 mM calcium chloride, manganese chloride, or zinc sulfate for three trials. (**E**) Cfu/mL after the eight-hour incubations normalized to the cfu/mL immediately prior to the incubations for the experiments from panel **D**. All graphs show mean values and standard deviations. Double or triple asterisks indicate p<0.05, 0.01, or 0.001, respectively, and “ns” indicates p>0.05. Asterisks over individual columns indicate differences compared to the control or wild type value. Horizontal bars indicate comparisons between mother and daughter cells.

Incubation in control YPD medium increased the relative retromobility frequency 1.1-fold, consistent with the rate of Ty1 retromobility [5]. Incubations in rich medium lacking glucose, YPD with 10 or 100 mM calcium chloride, or YPD with 0.5 M sodium chloride reduced retromobility by 1.3 to 4.9-fold (Fig. 3A). Cells doubled approximately once or twice during these treatments, so growth arrest did not account for the decreases (Fig. 3B). Retromobility was strongly inhibited in cells grown in YPD with 100 mM calcium chloride overnight for sorting mother and daughter cells (Fig. 3C). The relative inhibition was greater in mother cells, though, resulting in the absence of retromobility asymmetry between mothers and daughters (Fig. 3C). Therefore, calcium directly or indirectly, such as by induction of P-bodies, regulates this asymmetry.

To determine whether these results are specific to calcium or whether exposure to other divalent cations would produce similar results, we tested the impact of manganese and zinc on Ty1*his3AI* retromobility. Manganese has previously been reported to inhibit Ty1 reverse transcriptase, and zinc inhibits the HIV retroviral reverse transcriptase [37,38]. However, our strains were unable to grow in 10 mM manganese chloride or zinc sulfate, which is the lower of the calcium concentrations we used. Therefore, we repeated the eight-hour exposure experiments with 10 mM manganese chloride or zinc sulfate and calculated relative Ty1 retromobility frequencies to test for short-term effects on retromobility. Exposure to manganese chloride decreased retromobility 1.5-fold (Fig. 3D), but there was no increase in colony-forming units/ml (cfu/mL) (Fig. 3E), indicating that growth was arrested or cells were losing viability. Exposure to zinc sulfate increased retromobility 4.7-fold (Fig. 3D), but had a toxic effect on cells, since there was a sharp decrease in cfu/mL (Fig. 3E). These results indicate that not all divalent cations have the same impact on retromobility, but toxic effects of the high concentrations of manganese and zinc complicate interpreting their specific effects on Ty1.

### Retromobility asymmetry is also observed between NQ and Q stationary phase cells and regulated by pH homeostasis, *LSM1*, and calcium

**Figure 4:**
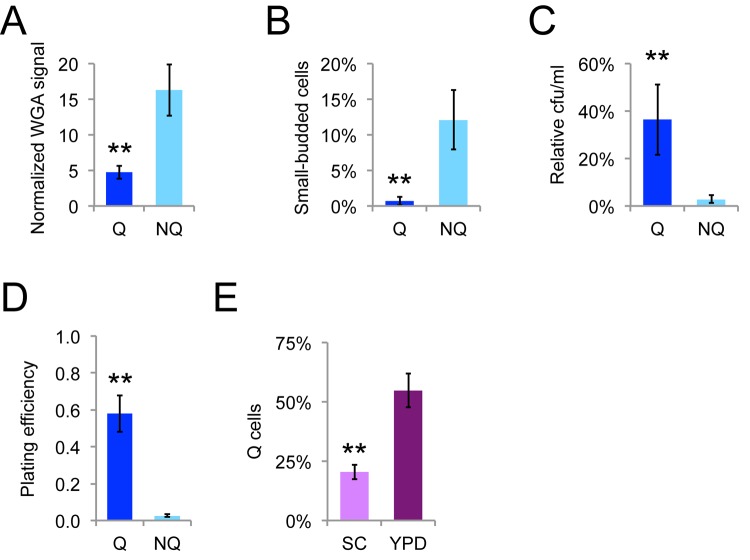
**Fractionated Q and NQ cells show expected phenotypes.** The relative level of WGA Alexa-488 staining of bud scars relative to unstained cells (**A**), percentage of cells with buds less than or equal to 50% the size of mothers (**B**), that could form colonies on YPD medium after heat shock at 52˚C for 20 minutes (**C**), or that could form colonies on YPD medium without treatment (**D**) for Q and NQ cells fractionated from populations grown for seven to eight days at 20˚C. (**E**) Proportion of fractionated Q cells in SC or YPD medium determined by counting cells by microscopy. Mean and standard deviations for seven or ten trials are shown and double asterisks indicate p<0.01 compared to NQ cells.

The final cell divisions as stationary phase yeast cells transition to quiescence are very asymmetric [18], giving rise to quiescent (Q) cells and nonquiescent (NQ) cells. Q cells are typically newborn daughters and young mothers and NQ cells comprise a heterogeneous subpopulation of cells that have not adapted properly for stationary phase that includes most of the older mother cells [19]. Ty1 mRNA has been reported to be in a protein-bound state in Q cells, but a protein-free state in NQ cells [20], raising the possibility that Ty1 is differentially regulated in these cells types. We fractionated NQ and Q cells by density-gradient centrifugation and verified that NQ cells were on average older, more likely to have small buds (indicating entry into S phase), more sensitive to heat stress, and the fraction of NQ cells that were able to grow into colonies when spread onto fresh solid medium (plating efficiency) was lower, as expected from previous work (Fig. 4A-D) [19]. A greater proportion of Q cells formed in YPD medium compared to synthetic complete (SC) medium (Fig. 4E), consistent with the negative impact of acetic acid accumulation on lifespan in SC medium [39].

**Figure 5:**
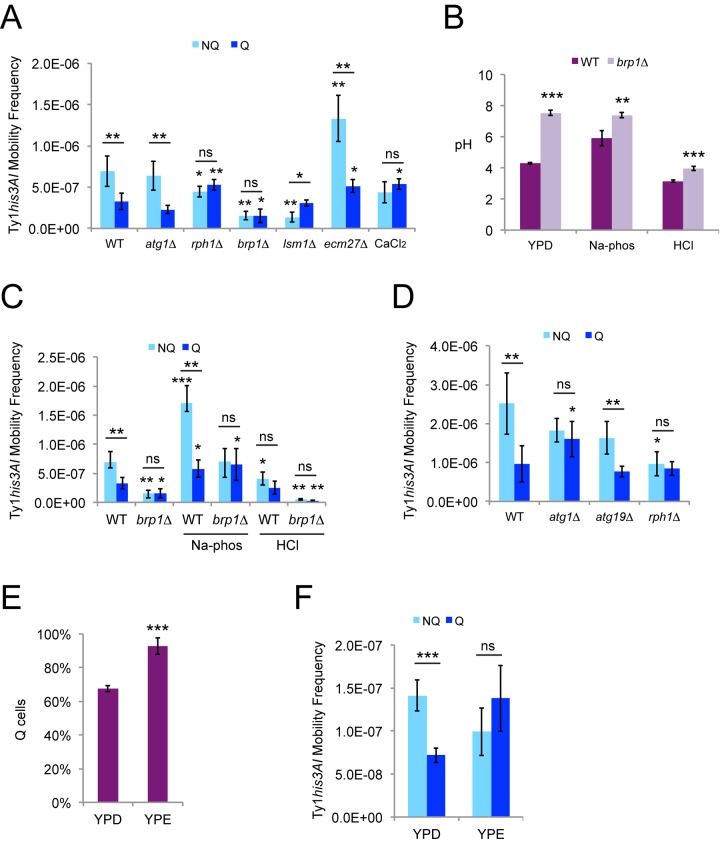
**Ty1 retromobility asymmetry between quiescent and nonquiescent stationary phase cells also depends on *LSM1*, calcium, and pH homeostasis.** (**A**) His^+^ frequencies in stationary phase cells grown in YPD and fractionated into quiescent (Q) and nonquiescent (NQ) subpopulations for the indicated genotypes or with chronic exposure to 100 mM calcium chloride. Results from three to six trials are shown. (**B**) Final (stationary phase) medium pH for Q and NQ cells from wild type or *brp1∆* mutants for cells grown in control YPD medium, or YPD medium with an initial pH of 7.1 or 4.1 due to addition of 20 mM sodium phosphate (Na-phos) or 30 mM hydrochloric acid (HCl), respectively. Data are from three to five trials each. (**C**) His^+^ frequencies in Q and NQ cells for the strains and control or alternative media conditions from panel **B**. Control wild type data are the same as in panel **A**. (**D**) His^+^ frequencies in Q and NQ cells fractionated from the indicated strains after growth in SC medium. Results are for three to five trials. (**E**) The proportion of Q cells after fractionation of cells grown to stationary phase in YPD or YP + 2% ethanol (YPE) for three trials. (**F**) His^+^ frequencies in Q and NQ cells isolated after growth in YPD or YPE for three trials. All graphs show mean values with standard deviation, and symbols for statistical significance are as for Fig. 2.

Ty1 retromobility was significantly higher in NQ cells than Q cells (Fig. 5A). We tested a subset of the mutants from the mother-daughter cell sorting experiments, chronic exposure to high calcium, and *ecm27∆* mutants defective for import of calcium during stationary phase [40] for changes in retromobility asymmetry between the NQ and Q cell subpopulations. Retromobility asymmetry was not observed in *brp1∆* or *rph1∆* mutants, or with high calcium exposure (Fig. 5A). Lack of retromobility asymmetry in the *rph1∆* mutants and cells exposed to calcium chloride resulted from modest decreases in NQ cell frequencies (not a significant decrease for calcium chloride) and modest increases in Q cell frequencies. While these frequency changes were relatively small and may seem of limited biological significance, the absence of asymmetry is a meaningful result. Overall retromobility was substantially decreased in *brp1∆* mutants, but the inhibition disproportionately affected NQ cells to abolish asymmetry between the two cell types (Fig. 5A). In contrast, NQ cell retromobility was strongly inhibited in *lsm1∆* mutants but there was no change in Q cell retromobility frequencies, leading to higher retromobility in Q cells and a reversal of the asymmetry (Fig. 5A). High calcium did not reduce retromobility overall (Fig. 5A), in contrast to the mother-daughter sorting experiments (Fig. 3C). Cells defective for accumulation of calcium during stationary phase (*ecm27∆*) maintained retromobility asymmetry between NQ and Q cells, but the overall retromobility frequencies increased (Fig. 5A).

Buffering of the medium was required for the loss of retromobility asymmetry in *brp1∆* mutants in the mother-daughter cell sorting experiments but not in stationary phase cells, so we tested if there was a difference in the final medium pH in stationary phase for the wild type strain compared to the *brp1∆* mutants. Stationary phase yeast cells acidify their medium [39], and medium pH decreased from 5.9 ± 0.11 to 4.3 ± 0.063 for the wild type strain grown in standard YPD (Fig. 5B). However, the final medium pH for *brp1∆* mutants was 7.5 ± 0.16. Increasing or decreasing the initial pH of YPD to 7.1 ± 0.14 using sodium phosphate buffer or to 4.1 ± 0.071 using hydrochloric acid changed the final medium pH to 5.9 ± 0.48 or 3.2 ± 0.058 for the wild type strain, respectively (Fig. 5B). The *brp1∆* mutants maintained a significantly higher pH in each of these treatments (Fig. 5B).

Increasing or decreasing the initial pH of YPD medium as described above increased or decreased Ty1 retromobility in the fractionated NQ and Q cells, respectively (Fig. 5C). Greater relative changes were observed in the NQ cells, which moderately increased the NQ to Q cell ratio for retromobility from 2.1 in standard YPD to 3.0 in the high pH condition and decreased the ratio to 1.6 in the low pH condition. No retromobility asymmetry was observed for *brp1∆* mutants in any of the conditions, and the treatments led to greater effects on overall retromobility frequencies relative to the control condition in *brp1∆* mutants (Fig. 5C), consistent with the defect in Pma1p activity compromising the ability of these cells to respond to pH changes. These data further emphasize the sensitivity of Ty1 retromobility to pH changes.

Defects in autophagy do not impair survival during stationary phase when cells are grown in YPD, but do reduce chronological lifespan when cells are grown in synthetic medium, which is correlated with a greater induction of autophagy at the start of stationary phase in SC medium [41]. Retromobility asymmetry between fractionated NQ and Q cells was not observed for *atg1∆* strains grown in synthetic complete (SC) medium because of an increase in retromobility in Q cells (Fig. 5D), in contrast to what was observed for *atg1∆* mutants in YPD (Fig. 5A). We also tested *atg19∆* mutants lacking a protein shown to target Ty1 Gag for autophagy [14], but asymmetry was still observed in these mutants (Fig. 5D), raising the possibility that a different factor could target Ty1 proteins for autophagy during stationary phase. There was no retromobillity asymmetry observed in *rph1∆* mutants in SC, as in YPD, but loss of asymmetry was only due to a decrease in NQ cell retromobility for SC (Fig. 5D). These results indicate that the influence of autophagy on retromobility asymmetry is dependent on the media conditions.

The NQ cells in our experiments were on average older than the Q cells (Fig. 4A), which means that the retromobility asymmetry between NQ and Q cells could reflect the asymmetry between mothers and daughters. We tested whether retromobility asymmetry would still be observed between NQ and Q cells if the proportion of Q cells were substantially increased, which would result in more mother cells in the Q cell fraction. We expected this to diminish NQ-Q cell retromobility asymmetry if it results from mother-daughter asymmetry or to have little effect on asymmetry if it results from specific changes in Ty1 regulation in NQ and Q cells. We grew cells to stationary phase in YP + 2% ethanol medium (YPE) to try to obtain a greater proportion of Q cells, since growth in nonfermentable carbon sources has been shown to extend lifespan during aging at 30˚C and 20˚C [39,42,43]. Cells grew more slowly in YPE and were incubated for 10-11 days at 20˚C before fractionations were performed, as compared to seven days in YPD. After growth in YPE, 93% of cells were Q cells, compared to 68% Q cells in YPD (Fig. 5E). There was no retromobility asymmetry between cell types in YPE (Fig. 5F), which is consistent with the NQ-Q cell asymmetry resulting from mother-daughter asymmetry.

Since pH was found to influence Ty1 retromobility in stationary phase cells (Fig. 5C), we also measured the initial and final medium pH for cells in SC and YPE medium. For SC, the initial and final values were 4.3 ± 0.015 and 2.7 ± 0.081, respectively. These values are much lower than those for YPD medium, and the low final medium pH is consistent with the accumulation of acetic acid during aging in SC [39]. However, overall frequencies were not decreased in SC medium compared to YPD medium. The initial and final pH values for YPE were 6.8 ± 0.012 and 7.52 ± 0.030, respectively, which were higher than those for YPD. Overall Ty1 retromobility was not very different between YPD and YPE (Fig. 5F), despite the higher pH. While Ty1 is sensitive to pH changes, these data indicate that pH is not always the major determinant of Ty1 retromobility frequencies.

### Exposure to high levels of calcium decreases total Gag levels and the proportion of processed p45-Gag

**Figure 6:**
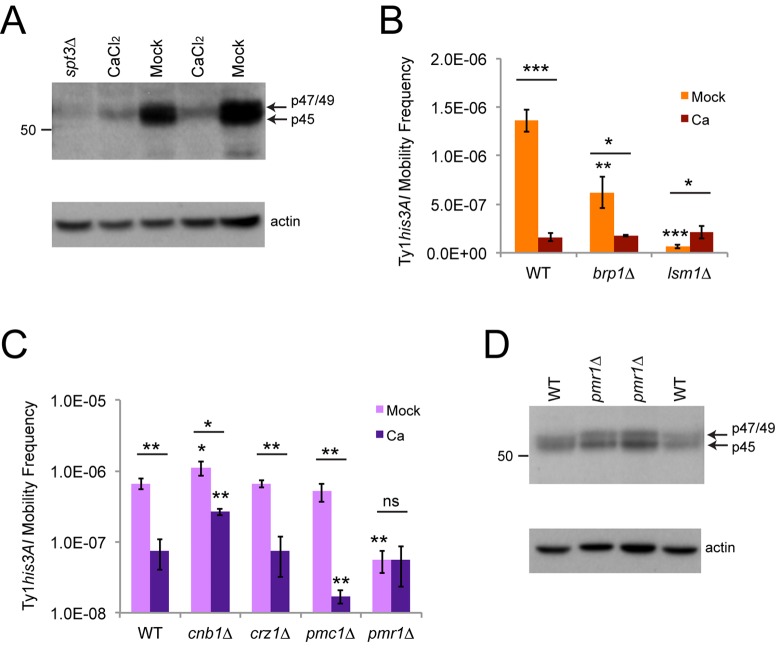
**Calcium treatment reduces Gag levels and masks or suppresses the effects of *brp1∆* or *lsm1∆* mutations.** (**A**) Western blot of protein extracts from cells grown to exponential phase in YPD with or without 100 mM calcium chloride probed with an affinity-purified polyclonal Gag antibody (upper panel). Arrows indicate the migration of the different forms of Gag, and the number on the left indicates the migration of a protein size standard in kilodaltons (kD). Note that the blot was moderately overexposed to better show the weak signal for calcium-treated cells. The lower panel shows the same blot stripped and reprobed with a beta-actin antibody as a loading control. The extract from an *spt3∆* mutant was used as a control for low expression. (**B**) His^+^ frequencies for mid-exponential phase cells of the indicated genotypes grown in YPD medium without (Mock) or with 100 mM calcium chloride (Ca). Data are for three trials. Horizontal bars indicate comparisons between treated and untreated cells. (**C**) Same as for panel **B** for three trials with a different set of mutant strains. Note that a log scale is used for the y-axis because of the large differences in frequencies. (**D**) Western blot for Gag in wild type (WT) and *pmr1∆* mutant strains, as described for panel **A**. All data in graphs are mean values with standard deviation. Asterisks over individual columns indicate comparisons to wild type treated or untreated cells. Asterisks indicate significant differences as for Fig. 2.

Since inhibition of Ty1 retromobility by calcium has not previously been investigated, we measured Ty1 Gag levels by Western blotting in cells exposed to calcium to determine if inhibition was occurring at an early or late step in the retromobility cycle. Three forms of Gag have been detected by Western blotting: protease-processed p45, unprocessed p49, and a phosphorylated form that migrates at a position intermediate to p49 and p45 that accumulates in cells arrested in G1 with mating pheromone [8,21]. We refer to the latter form as p47-Gag, due to its intermediate mobility, and its presence has been correlated with inhibition of intermediate steps in the retromobility cycle [21]. Gag signal frequently appears as a doublet interpreted as representing p45 and p49, but previous work has shown that the slower migrating form detected in the strain background we used is not p49, indicating that it may represent p47-Gag [9], so we refer to the upper band on our Westerns as p47/49-Gag. Total Gag levels were reduced 5.5-fold in cells grown to exponential phase in YPD + 100 mM calcium chloride (n=6, p<0.001), indicating that inhibition occurs early during the retromobility cycle (Fig. 6A). The majority of the Gag signal in cells exposed to calcium was the slower migrating p47/p49-Gag, and only 6.8 ± 4.5% of total Gag was p45 in treated cells, compared to 76 ± 9.2% in mock-treated mid-to-late exponential phase cells (n=6, p<0.001).

High calcium, *brp1∆* mutants (in buffered medium), and the mRNA-decay mutants tested all inhibited overall Ty1 retromobility with a greater relative impact on mother cells to prevent retromobility asymmetry between mothers and daughters, raising the possibility that they could work through similar mechanisms (Figs. 2 and 3). However, each of these factors had different types of effects on NQ and Q cells to take away retromobility asymmetry between these subpopulations of cells (Fig. 5A). Two prior studies have shown that mutants deficient for several mRNA decay/core P-body factors, including *lsm1∆* and *pat1∆* mutants, have moderately reduced Ty1 Gag protein levels [10,11], while one prior study has shown that *brp1∆* mutants have wild type Gag levels [30]. We found that Ty1 retromobility frequencies were the same in wild type, *brp1∆*, and *lsm1∆* mutants with chronic exposure to high calcium (Fig. 6B), showing that calcium can mask/suppress the effects of these mutants. This indicates that there may be some interaction between the effects of these factors on Ty1.

We further tested whether factors involved in calcium signaling and transport contributed to the inhibition of Ty1 by calcium. Calcineurin is a highly conserved phosphatase activated by calcium/calmodulin that regulates many proteins, including activating the yeast Crz1p transcription factor that increases expression of approximately 100 genes in response to increased calcium [44]. Two example targets of Crz1p are the Pmc1p and Pmr1p cation pumps that concentrate calcium in the vacuole, which stores most intracellular calcium in yeast, and the ER, respectively [44]. We measured Ty1 retromobility in *cnb1∆* mutants lacking a non-essential regulatory subunit of calcineurin, as well as *crz1∆*, *pmc1∆*, and *pmr1∆* single mutants with and without exposure to 100 mM calcium chloride. In mock-treated cells, the *crz1∆* and *pmc1∆* strains had wild type retromobility, while *cnb1∆* strains had a very modest 1.7-fold increase in retromobility (Fig. 6C). The *pmr1∆* mutants exhibited a 12-fold decrease in retromobility, consistent with a prior investigation of *pmr1* mutants [37], and a log scale was used for the y-axis to more clearly show the low frequency in these mutants. Calcium had the same inhibitory effect on retromobility in *crz1∆* mutants, a moderately diminished effect in *cnb1∆* mutants, a stronger effect in *pmc1∆* mutants, and no further effect on mobility in *pmr1∆* mutants (Fig. 6C). These results indicate that the effect of calcium on Ty1 is partially dependent on calcineurin, but not Crz1p, and sensitive to the presence/absence of the Pmc1p and Pmr1p cation pumps. Previous work has shown that the absence of Pmr1p does not substantially reduce levels of Gag [37], which we confirmed in our strain background (Fig. 6D), demonstrating that the inability of calcium to further inhibit Ty1 in these mutants is not because *pmr1∆* mutants already have low levels of Gag.

### Mother cells have higher total Gag levels than daughters

**Figure 7:**
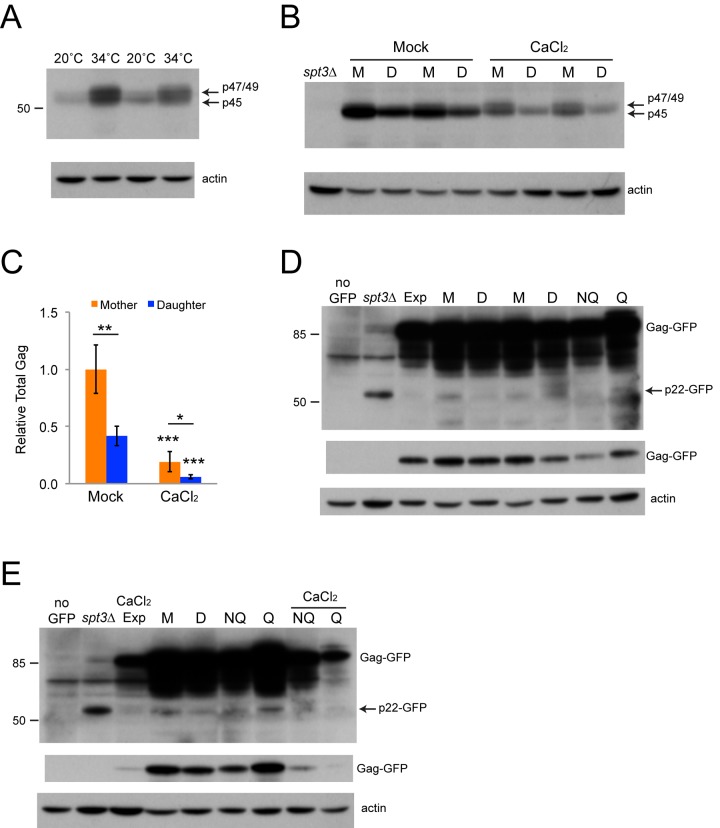
**Mother cells have increased levels of Gag.** (**A**) Western blot of protein extracts from cells grown in YPD to late exponential phase at the indicated temperatures. Upper panel shows the blot probed with Gag antibody and the lower panel shows the blot stripped and reprobed with beta-actin antibody. Arrows indicate the migration of the alternative forms of Gag, and the number to the left shows the migration of a 50 kD protein size standard. (**B**) Western blot for Gag protein present in extracts of sorted mother (M) and daughter (D) cells grown in YPD without or with 100 mM calcium chloride, as described for panel **A**. An extract from an *spt3∆* strain was used as a control for low Gag expression. (**C**) Quantification of the Gag signal normalized to beta-actin from Western blots of mother and daughter cell extracts grown with or without 100 mM calcium chloride. Data are mean and standard deviation values for three trials. Horizontal bars indicate comparisons between mothers and daughters. Asterisks over individual columns indicate comparisons to mock treatment. Symbols for statistical significance are as for Fig. 2. (**D-E**) Westerns blots of extracts prepared from mother (M), daughter (D), nonquiescent (NQ), quiescent (Q), and exponential phase (Exp) cells expressing a Gag-GFP fusion protein, or a control strain lacking the fusion protein (no GFP). Upper two blot panels in each case were probed with a GFP antibody, with the top image being overexposed to show p22-GFP (arrow). Middle image shows a shorter exposure for Gag-GFP, and bottom image shows the blot stripped and reprobed with beta-actin antibody. Some extracts were prepared from cells grown in 100 mM calcium chloride for panel **E**, as indicated. Migration of 85 and 50 kD size standards is indicated to the left for the top image.

We previously used two-photon fluorescence microscopy to show that there was no substantial difference in the overall concentration of a Gag-GFP fusion protein between mothers and daughters, though individual cells with the highest Gag-GFP concentrations tended to be in the mother cell populations [5]. We decided to confirm this and to test if there might be different forms of Gag in mothers and daughters by Western blotting protein extracts from sorted mother and daughter cells. First, we tried to determine whether the upper band in the Gag doublet we observed represented p47 or p49-Gag. Since Ty1 protease is temperature sensitive [45] we grew cells to late exponential phase at 34˚C to inhibit protease without treating the cells so harshly that there might be other confounding effects on Ty1. Gag from cells grown at 34˚C still formed the same doublet without any new bands, but a greater proportion of the signal was from the upper band and only 50 ± 2.3% of the signal was from p45-Gag, compared to 80 ± 4.0% for cells grown at 20˚C (n=3, p<0.001). This supports the interpretation that the upper band includes unprocessed p49-Gag, and we therefore continue to refer to the upper band as p47/p49-Gag. Unexpectedly, cells grown at 34˚C had moderately increased Gag levels (Fig. 7A). For protein extracts from mother and daughter cells, Gag signal was virtually all from p45-Gag, and we were surprised to find that mother cells had 2.4-fold higher levels of Gag than their daughter cells (Figs. 7B and 7C).

Mother and daughter cell extracts from cells grown in YPD + 100 mM calcium chloride had significantly less total Gag protein, as expected (Figs. 7B and 7C). Total Gag was 3.0-fold higher in treated mothers than their daughters, similar to the fold change observed for mock-treated cells. Interestingly, there was a decrease in the proportion of p45-Gag only in the treated mother cells, with virtually all Gag signal coming from p45-Gag in the treated daughter cells (Fig. 7B). Quantification of the signals in mother cells demonstrated that p45-Gag accounted for 96 ± 4.5% of total Gag in mock-treated mothers, compared to 72 ± 6.0% in calcium-treated mothers (n=3, p<0.001). This difference specifically in mother cells could account for the greater relative inhibition of Ty1 retromobility in mother cells compared to daughter cells with calcium treatment leading to loss of retromobility asymmetry.

Ty1 produces a dominant-negative form of Gag, p22, which inhibits retromobility at least partly by blocking proper VLP formation [12]. The antibody that we used to detect Gag does not reliably detect p22, so we utilized two congenic strains in our strain background harboring a single chromosomal Ty1 expressing a Gag-GFP fusion, with GFP fused at the processing site near the C-terminal end of Gag. This fusion was expected to result in expression of p22-GFP as well as Gag-GFP, since p22 is an N-terminally truncated form of Gag [12], allowing p22 detection using a GFP antibody. We included extracts from *spt3∆* derivatives of these strains on Western blots, since *spt3∆* mutants increase the level of p22 [12], as well as from a wild type strain that lacks the Gag-GFP fusion to confirm that we were able to detect p22-GFP. The p22-GFP signal was much weaker than Gag-GFP, was stronger in the *spt3∆* strains, and absent from the strain lacking a GFP fusion (Figs. 7D and 7E). We did not observe any consistent reduction in p22-GFP in mother cells relative to daughter cells that could account for retromobility asymmetry. We examined extracts from NQ and Q cells with and without treatment with calcium chloride and from cells grown to exponential phase with calcium chloride treatment, but we did not observe consistent decreases in p22-GFP in NQ cells or increases in p22-GFP in the treated cells (Figs. 7D and 7E). We also found that Gag-GFP was only reduced 1.6-fold in daughters relative to mother cells (n=3, p<0.01), in contrast to the 2.4-fold decrease seen for wild type Gag, indicating that the fusion protein may not be as asymmetrically distributed. These results indicate that asymmetry in p22 expression is not likely responsible for retromobility asymmetry, provided that the GFP fusion reliably reflects wild type p22 distributions.

### Ty1 is inhibited during the transition to stationary phase and decreases the fitness of proliferating cells

**Figure 8:**
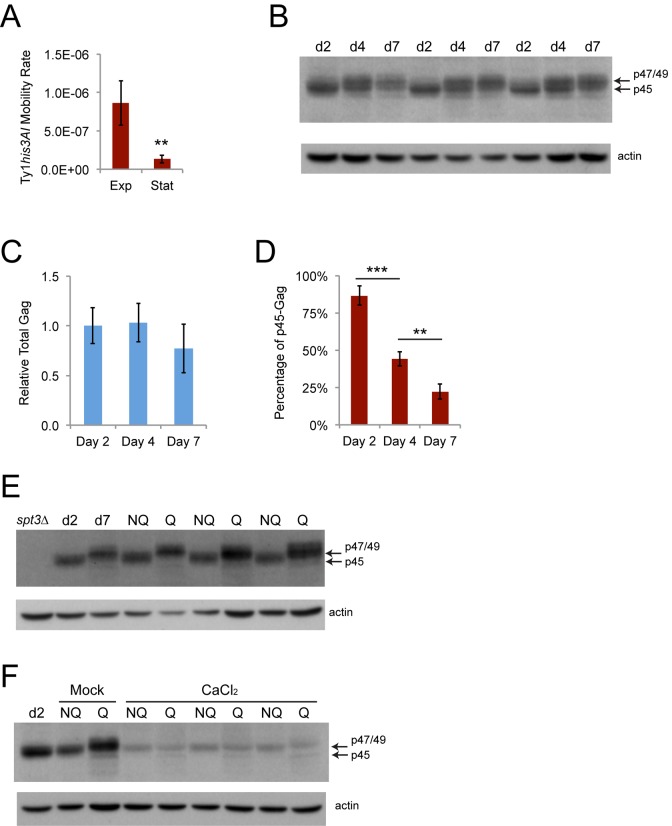
**Ty1 retromobility and the proportion of mature p45-Gag decrease as cells transition to stationary phase.** (**A**) His^+^ rates per cell generation for cells grown to mid-exponential (Exp, two days) or early stationary phase (Stat, four days). Data are for five trials. (**B**) Western blot probed with Gag antibody (upper panel) or reprobed with beta-actin antibody as a loading control (lower panel) for protein extracts prepared from cells grown for two days to exponential phase (d2), four days to early stationary phase (d4), or seven days to stationary phase (d7). Arrows indicate the migration of the different forms of Gag. (**C**) Quantification of Gag levels normalized to beta-actin in cells grown for the indicated number of days. Data are for three trials. (**D**) Quantification of the proportion of the total Gag signal that was comprised of p45 for cells grown for the indicated number of days for three trials. Data are from three trials. Horizontal bars indicate comparisons between days two and four or days four and seven. (**E-F**) Western blots for Gag and beta-actin as described for panel **B** for cell extracts prepared from exponential phase cells (d2, *spt3∆*), stationary phase cells (d7), or fractionated NQ and Q cells grown in YPD without (panel E and panel F Mock) or with (CaCl_2_) 100 mM calcium chloride. Graphs show mean values with standard deviation. Asterisks indicate significant differences as for Fig. 2.

Retromobility frequencies were much lower for the experiments using stationary phase cells than for the experiments sorting mother and daughter cells, which analyzed late exponential phase cells, with a 6.2-fold decrease in the average retromobility frequency of NQ cells compared to mother cells for wild type strain JC3787 (Figs. 2 and 5). The Ty1 retromobility rate per cell generation measured through fluctuation tests was 6.5-fold lower in cells grown to early stationary phase (four days at 20˚C) compared to mid-exponential phase (two days at 20˚C) (Fig. 8A). Total Gag levels were equivalent at mid-exponential phase (day two) and early stationary phase (day four), and were modestly but not significantly lower at the point at which we fractionated NQ and Q cells (day seven) (Figs. 8B and 8C). More strikingly, there was a steady decrease in the proportion of p45-Gag over this time course (Figs. 8B and 8D). We then found that virtually all the Gag signal in NQ cells is from p45, while virtually all the Gag signal for Q cells is from p47/49 (Fig. 8E). There was no substantial difference in the total amount of Gag between the NQ and Q cells, though. Treatment with calcium chloride substantially reduced total Gag in NQ and Q cells, as expected, but also resulted in p47/49-Gag being the major form of Gag in NQ cells (Fig. 8F). Accumulation of immature and inactive forms of Gag during stationary phase and in Q cells could account for the reduction in overall mobility and contribute to NQ-Q cell retromobility asymmetry.

**Figure 9:**
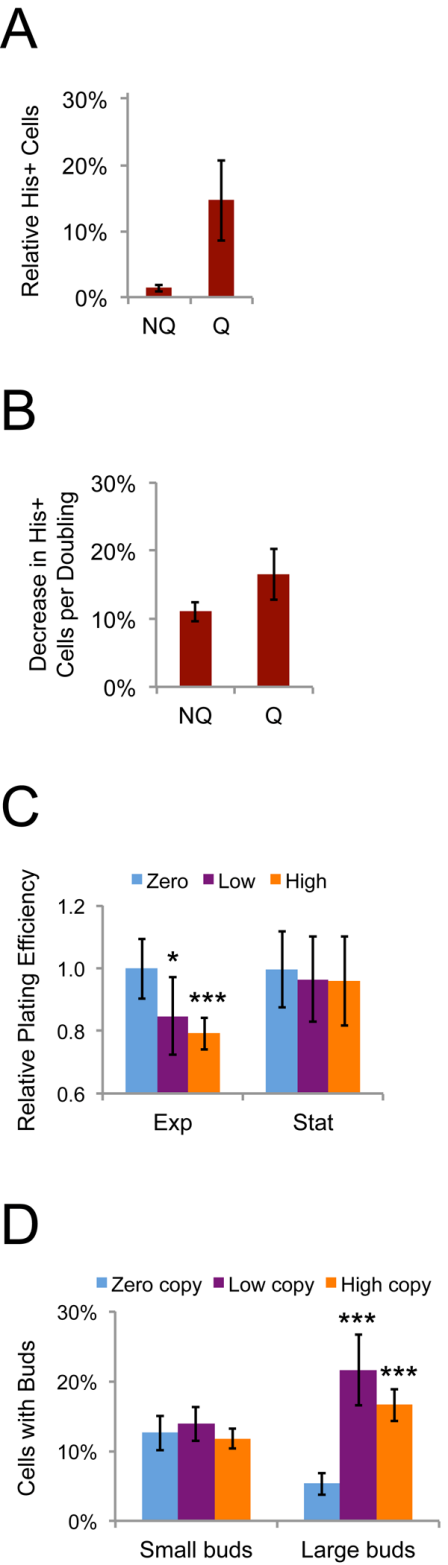
**Ty1 reduces the reproductive potential of dividing cells.** (**A**) Proportion of His^+^ cells in NQ or Q cell populations regrown in fresh medium at 30˚C overnight expressed as a percentage of the proportion of His^+^ cells in the populations prior to regrowth. (**B**) Relative decrease in the frequency of His^+^ cells per cell doubling in the populations regrown overnight for the data in panel **A**. (**C**) Plating efficiencies for mid-exponential (Exp, two days) and early stationary phase (Stat, four days) populations of *S. paradoxus* strains with zero, one to three (low), or about 20 (high) chromosomal copies of Ty1 grown at 20˚C and normalized to the values for the zero copy strain. Data are from five to nine trials. Three low copy and three high copy strains were used. (**D**) Percentages of cells with buds in strains with the indicated Ty1 copy number grown at 20˚C for seven days, determined by light microscopy. Buds less than or more than 50% the size of the mother cells were scored as small or large, respectively. Three low copy and three high copy strains were used and results are from five to six trials. All data are mean values with standard deviation. Single or triple asterisks indicate p<0.05 or 0.001, respectively.

Lower Ty1*his3AI* retromobility frequencies in stationary phase cells compared to exponential phase cells was unexpected, since the *his3AI* assay measures the cumulative frequency of Ty1 retromobility events, unless Ty1 retromobility during exponential phase was associated with senescence or cell death. As a first test of whether new Ty1 retromobility events would be correlated with reduced cell proliferation, we examined whether cells with new Ty1 insertions would be out-competed by cells without new insertions. The His^+^ frequency (Ty1 retromobility frequency) of fractionated NQ and Q cell populations was measured, and then these cells were grown in fresh medium overnight at 30˚C to inhibit new retrotransposition events. If cells with and without new Ty1 insertions (His^+^ and His^-^ cells, respectively) grew equally well overnight, we expected the proportion of His^+^ cells in the population to remain stable. In contrast, the proportion of His^+^ cells in the population substantially decreased after overnight growth for both cell types (Fig. 9A). Though the decrease was greater for NQ cells, this resulted from NQ cells undergoing more rounds of cell division, since a low percentage of the initial NQ cells were able to resume proliferation in the fresh medium (Fig. 4D). The decrease in the proportion of His^+^ cells was similar for both cell types when normalized to the number of cell doublings during the overnight growth (Fig. 9B).

We further tested whether Ty1 could reduce reproductive potential of cells by comparing an *S. paradoxus* strain completely lacking Ty1 elements to three derivatives with low Ty1 copy number (1-3 chromosomal copies) and three derivatives with high Ty1 copy number (~20 chromosomal copies) [46]. The fraction of cells that could form colonies on fresh medium (plating efficiency) was significantly reduced in mid-exponential but not stationary phase populations for all strains with Ty1 compared to the zero copy parent strain, supporting a negative influence of Ty1 expression or mobility on reproductive potential (Fig. 9C). Though there was no decrease in reproductive potential of stationary phase cells with Ty1, we observed that stationary phase cells with Ty1 were more likely to have large buds (G2/M phase cells) than the zero Ty1 copy cells (Fig. 9D), indicating that Ty1 expression or mobility affects cell cycle progression/arrest. Since the effects in Fig. 9C and 9D were observed in strains with both low and high genomic copy numbers of Ty1, they appear to occur over a range of Ty1 expression levels. The negative influence of Ty1 on fitness of proliferating cells could therefore account for the decreased retromobility frequencies in stationary phase cells, since exponential phase cells in which Ty1 is active may stop dividing or be outcompeted as the population continues towards stationary phase.

### Q cells could not be isolated from a Ty1-less *S. paradoxus* strain background

**Figure 10:**
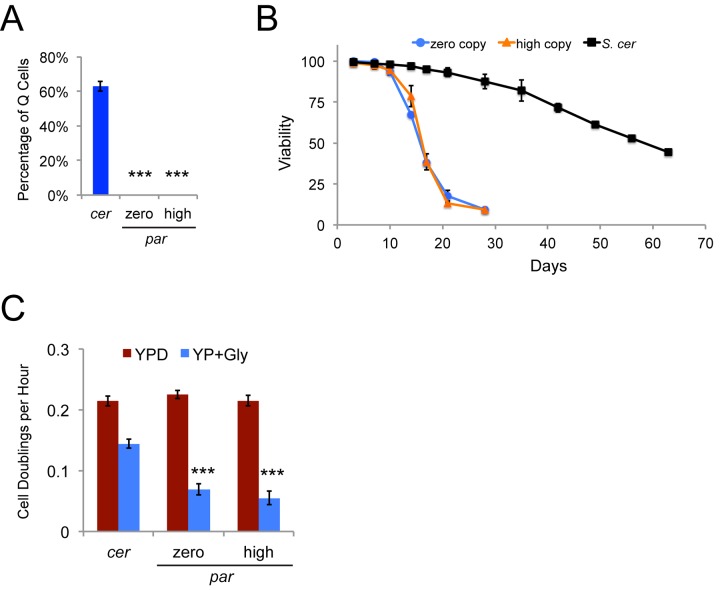
**A Ty1-less *S. paradoxus* strain background does not yield a Q cell fraction and has a short chronological lifespan.** (**A**) Proportion of Q cells from stationary phase cultures following density gradient fractionation of the *S. cerevisiae* (*cer*) and *S. paradoxus* (*par*) zero and high Ty1 copy strains. Data are from three trials. (**B**) Chronological lifespan at 20˚C determined by measuring cell viability through trypan blue dye exclusion for three trials with a wild type *S. cerevisiae* strain (*S. cer*) and the zero and high Ty1 copy *S. paradoxus* strains. (**C**) Growth rates (cell doublings per hour) for three trials of a wild type *S. cerevisiae* strain (*cer*) and four trials of the zero and high Ty1 copy *S. paradoxus* strains (*par*) grown in rich medium with glucose (YPD) or glycerol (YP + Gly) as a carbon source. All data are mean values with standard deviation. Triple asterisks indicate p<0.001.

We fractionated the *S. paradoxus* strains with zero or varying copy numbers of Ty1 to determine whether the presence of Ty1 would alter the proportion of stationary phase cells that appropriately enter quiescence, but no Q cell fractions were obtained from the strains in this background. This is a characteristic of the strain background and not a phenotype related to Ty1, since both the original zero copy strain and the three high Ty1 copy derivatives used for the prior set of experiments failed to yield Q cell fractions (Fig. 10A). In contrast, approximately 63% of stationary phase cells were Q cells for a wild type Ty1*his3AI S. cerevisiae* strain (Fig. 10A). We compared the chronological lifespans of the zero and high Ty1 copy *S. paradoxus* strains to the wild type *S. cerevisiae* strain aged at 20˚C in YPD medium, since Q cells have longer lifespans than NQ cells [18]. The median chronological lifespan (days to 50% viability) was 58.7 days for the *S. cerevisiae* strain compared to 15.8 and 16.1 days for the zero and high Ty1 copy *S. paradoxus* strains, respectively (p<0.001 for zero or high Ty1 copy strains compared to *S. cerevisiae* strain) (Fig. 10B). Mitochondrial activity has been shown to be important for formation and function of Q cells [20,47]. We grew the wild type *S. cerevisiae* strain and the zero and high Ty1 copy *S. paradoxus* strains in rich medium containing either glucose or glycerol as a carbon source to compare their growth rates when cells could use fermentation (glucose) or had to use aerobic respiration (glycerol) to utilize the carbon source. All strains had similar growth rates in glucose containing medium (YPD), but the zero and high Ty1 copy *S. paradoxus* strains exhibited less than half the respiratory growth rate of the *S. cerevisiae* strain in glycerol medium (YP + Gly) (Fig. 10C). Growth rates were measured for the first 32 hours of growth, but the *S. paradoxus* strains never doubled more than 3-4 times in glycerol medium even after incubation for seven days at 20˚C. These strains are therefore defective in some aspect of respiratory growth. While it is possible that the *S. paradoxus* strains form Q cells that cannot be fractionated by the method we used, the shorter lifespans and defective respiratory growth are consistent with a defect in either the formation or function of Q cells.

## DISCUSSION

We have shown that Ty1 retromobility asymmetry between mother and daughter cells as well as NQ and Q cells depends on mRNA decay factors, pH homeostasis, and can be abolished by exposure to high calcium. Total Ty1 Gag levels were increased in mother cells compared to their daughters, which could account for increased retromobility in mothers, but is in contrast to our previous results from analysis of a Gag-GFP fusion protein by microscopy [5]. The GFP fusion for that construct is at the processing site for Gag, which prevents protease processing of Gag-GFP, and we found that nearly all Gag in cells in our mother-daughter sorting experiments is the processed p45 form. There was a higher level of Gag-GFP in mothers compared to daughters as measured on Western blots, but the asymmetry was not as pronounced as for wild type Gag. Perhaps the lack of processing or some other aspect of the fusion diminishes its asymmetric distribution. There was a steady decrease in mature p45-Gag as cells transitioned from exponential phase to stationary phase without a substantial decrease in total Gag. NQ cells contained p45-Gag, while Q cells contained unprocessed p49 or phosphorylated p47. Accumulation of immature p49-Gag reflects inhibition of VLP formation or maturation [8], and accumulation of p47 is associated with VLPs that have reduced levels of Pol protein and reduced reverse transcriptase activity [21]. An increased proportion of either of these forms of Gag could account for reduced retromobility during stationary phase and in Q cells. Exposure to high calcium reduced total Gag levels and increased the proportion of p47/49-Gag particularly in mothers and NQ cells, consistent with the absence of retromobility asymmetry in treated cells. Many factors regulating retrotransposition and impacts of retrotransposons are conserved between Ty1 and mammalian retrotransposons [2,8,24], and our results may prove relevant to further understanding the potential impact of mammalian retrotransposons on aging.

The contribution of mRNA decay/P-body factors Lsm1p, Pat1p, and Upf3p to retromobility asymmetry is consistent with asymmetry in processing of Ty1 mRNA or formation of Ty1 retrosomes, since retrosome formation is inhibited in *lsm1∆* and *pat1∆* mutants [10]. Retrosomes were previously observed to be more abundant in budding mother cells than cells that were not dividing and absent when P-body formation was induced [10]. Formation of P-bodies could prevent P-body proteins from interacting with Ty1 protein/RNA and thereby inhibit retromobility. Loss of mRNA decay factors prevented retromobility asymmetry by more strongly reducing retromobility in mother and NQ cells, indicating that these factors are facilitators of some step(s) in retromobility particularly in these cell types. There may be differences in the functions of P-body and mRNA decay factors in mothers versus daughters that could lead to retromobility asymmetry, which is supported by the observation that P-bodies localize to the bud site of cells prior to cell division and are transported into daughter cells [48]. Mammalian L1 retrotransposons also interact with cytoplasmic RNA granules [49], and the yeast Ty1 system may provide a useful model for understanding the regulation and impact of those interactions.

Enrichment of Pma1p in mother cells relative to daughter cells is one of the earliest asymmetries observed during yeast replicative aging, and contributes to higher intracellular pH, reduced vacuole acidity, and reduced mitochondrial function in aging mother cells [13]. Reduced Ty1 retromobility in *brp1∆* mutants was complemented by expression of *PMA1*, and retromobility asymmetry was not observed for *brp1∆* mutant mother and daughter cells when medium was buffered to pH 7.1 (similar to the cytoplasmic pH of proliferating yeast cells [13]) or during stationary phase when cells normally acidify their medium [39]. Buffering may have limited the ability of daughters to concentrate protons and thereby changed some aspect of their cell function, since buffering led to an increase in retromobility only in mutant daughter cells to take away asymmetry. Improved autophagy in *brp1∆* mutants due to increased vacuole acidity is unlikely responsible for the absence of asymmetry, since loss of autophagy only abolished retromobility asymmetry in stationary phase cells grown in SC medium. Mitochondrial function and reactive oxygen species regulate Ty1 [50], so altered pH homeostasis may influence retromobility asymmetry through mitochondrial function. Alternatively, a posttranscriptional step in retrotransposition may be directly sensitive to cytoplasmic pH.

Growth in medium with a high calcium concentration took away retromobility asymmetry, while *ecm27∆* mutants defective for calcium accumulation in stationary phase [40] had elevated retromobility but maintained asymmetry. Exposure to high calcium levels substantially decreased overall retromobility frequencies in proliferating cells, but not in stationary phase cells. This could be due to the normal accumulation of calcium during stationary phase [40], which could at least partly account for reduced retromobility as cells transition to stationary phase. Calcium has been shown to inhibit retroviral reverse transcription [51], but we observed decreased total Gag levels and an increased proportion of p47/49-Gag in response to high calcium. Inhibition by calcium was only partly dependent on the calcium-responsive phosphatase calcineurin, and independent of the Crz1p transcription factor activated by calcineurin. Crz1p-independent functions of calcineurin, such as changes in sensitivity to pH changes or in secretory vesicle trafficking [44], therefore influence the impact of calcium on Ty1. Pmc1p transports calcium into the vacuole, the major site of calcium storage [44], and the greater inhibition by calcium in the absence of Pmc1p could be due to a higher cytoplasmic calcium concentration from lack of transport. The loss of the Pmr1p calcium transporter for the ER was previously reported to inhibit Ty1 without reducing Gag levels [37], and we found that calcium did not further inhibit Ty1 in these mutants. Ty1 Gag is translated at and translocated into the ER [9], and calcium is concentrated in the ER by Pmr1p [44]. The absence of Pmr1p may disrupt ER function in a way that inhibits Gag trafficking or stability, diminishing the possible impact of the reduction in total Gag and mature p45-Gag caused by high calcium levels. The absence of an additive influence on retromobility by treating *brp1∆* mutants with calcium may result from reduced uptake of calcium by cells with reduced Pma1p activity [52]. The moderate suppression of retromobility in *lsm1∆* mutants by calcium could result from defects in translation and translocation of Gag into the ER due to high calcium. It has been shown that stalling Gag translation and ER translocation can increase retrosome formation [9], which could partly compensate for reduced retrosome formation in *lsm1∆* mutants [10]. Alternatively, induction of P-bodies by high calcium could draw non-productively bound factors away from Ty1 mRNA to P-bodies in *lsm1∆* mutants.

Changes in the abundance and forms of Gag between cell types provides an explanation for retromobility asymmetry, but the mechanism(s) responsible for these changes in Gag remains to be determined. Asymmetry in the dominant-negative p22-Gag protein does not appear to be relevant to retromobility asymmetry, with the caveat that the GFP fusion form of p22 may not be as asymmetrically distributed as wild type p22, since we found that Gag-GFP was less asymmetrically distributed than wild type Gag. The accumulation of p47-Gag was originally observed in cells arrested in G1 by mating pheromone [21]. Our observation that virtually all Gag in Q cells is p47/49 is consistent with that result, since Q cells are arrested in G1. Interestingly, treatment of *S. cerevisiae* cells with mating pheromone results in calcium transport and accumulation in cells [53,54], and we observed that calcium increased the proportion of p47/49-Gag. It would be interesting to determine if G1 arrest itself or an influx of calcium is responsible for the increased proportion of posttranslationally modified Gag.

There is significant conservation of aspects of asymmetric cell division, quiescence, and the regulation of retrotransposons between yeast and mammals [2,17,55]. When some mammalian stem cells divide to form a daughter cell that begins to differentiate and a daughter cell that remains a stem cell, there is asymmetric retention of less functional mitochondria and damaged proteins in the more differentiated daughter cell, as well as an endoplasmic reticulum diffusion barrier between the two daughter cells [22,23], similar to what is observed in yeast. Stem cells cycle between proliferation and quiescence for appropriate renewal of tissues during aging, and factors such as autophagy, cytoplasmic RNA granules, and DNA damage/replication stress influence quiescence in both yeast and mammalian cells [56–62]. Yeast and mammalian retrotransposons are regulated by autophagy, DNA damage, and reactive oxygen species [2,24].

Endogenous retrotransposons are active in mammalian stem cells, induced pluripotent stem cells, and during early differentiation of neural progenitor cells as compared to neural stem cells [63–65]. The latter observation raises the possibility that there is retrotransposition asymmetry between stem and more differentiated cells in mammals. Our results support a model whereby retrotransposon mobility could contribute to heterogeneity of cells undergoing replicative aging and cycling between proliferation and quiescence, decreasing cellular fitness during proliferation and having a greater impact on cells that will not successfully re-enter the quiescent state. Retrotransposons could therefore contribute to the decline in function of stem cell pools over time by increasing variation in these cells. Failure of mechanisms that restrict retrotransposons during quiescence as cell populations age could exacerbate this effect and further reduce the pool of functional stem cells. Our observation that the *S. paradoxus* strain either does not form Q cells or forms Q cells with compromised function is particularly interesting, since we previously found that Ty1 could promote chronological lifespan in this strain background [46]. This ability of retrotransposons to improve the survival of cells not correctly responding to cues for growth regulation may be relevant to understanding the potential consequences of the frequent activation of retrotransposons in human tumors [66]. Future work will test these potential influences of retrotransposons on stem cells and tumor cells in higher organisms.

## MATERIALS AND METHODS

### Yeast strains and media

Standard yeast extract-peptone-dextrose (YPD) medium and synthetic complete (SC) medium containing 2% glucose were used to grow yeast strains. For some experiments, medium contained a final concentration of 10 or 100 mM calcium chloride, 0.5 M sodium chloride, 10 mM manganese chloride, 10 mM zinc sulfate, or glucose was omitted from the medium, as indicated in the figures. For high and low pH treatments, YPD medium was brought to a pH of 7.1 using a final concentration of 20 mM sodium phosphate buffer pH 7.8 and to a pH of 4.1 using a final concentration of 30 mM hydrochloric acid. A Ty-less strain of *S. paradoxus*, DG1768 (*MATα*, *his3-∆200hisG, ura3*) [67], and derivatives harboring low copy (1-3) or high copy (~20) number of chromosomal Ty1 elements that have been previously described [46] were used for some experiments. Most other experiments were conducted using strains JC3787 or JC3212, derivatives of BY4742 (*MAT*α, *his3Δ1*, *leu2Δ0*, *lys2Δ0*, *ura3Δ0*) or BY4741 (*MATa*, *his3Δ1*, *leu2Δ0*, *met15Δ0*, *ura3Δ0*), respectively, carrying a single chromosomal copy of a Ty1*his3AI* element [68]. Derivatives of JC3787 or JC3212 with single gene deletions were generated by one-step gene replacement using *atg1::kanMX*, *atg19::kanMX*, *brp1::URA3*, *ecm27::kanMX*, *hsp104::kanMX*, *lsm1::kanMX*, *rph1::kanMX*, *rsr1::kanMX*, *scs2::kanMX*, or *spt3::kanMX* PCR products and lithium acetate transformation. Transformants were selected for the ability to grow on medium lacking uracil or containing 200 µg/mL G418. PCR was used to verify the absence of the wild type allele and presence of the deletion allele, and at least two transformants were tested for each genotype. The *pat1∆* and *upf3∆* derivatives of JC3212 have been previously described [11]. Strains JC3566 (*MAT*α, *his3Δ1*, *leu2Δ0*, *lys2Δ0*, *ura3Δ0*, Ty1*-GFP-3566*) and JC3687 (*MAT*α, *his3Δ1*, *leu2Δ0*, *lys2Δ0*, *met15∆0*, *ura3Δ0*, Ty1*his3AI*, Ty1-*GFP-3566*) are congenic to JC3787 and have *GFP* fused to nucleotide 1,203 of Ty1 *gag* (*TYA1*) in a chromosomal Ty1 element [69] (kindly provided by Joan Curcio, Wadsworth Center, Albany, NY), creating a fusion protein with GFP at the Gag processing site near the C-terminal end of the protein.

### *PMA1* plasmid construction and transformation into yeast

A chromosomal region from 690 bp upstream to 416 bp downstream of the *PMA1* open reading frame was amplified with PCR primers that introduced *SpeI* and *SacII* restriction enzyme sites at the 5′ and 3′ ends of the product, respectively. Two independent clones of the PCR product were introduced as *SpeI*-*SacII* fragments into the pRS415 low-copy yeast shuttle vector, containing a *LEU2* marker gene. The *PMA1* plasmids and pRS415 were transformed into wild type and *brp1::URA3* yeast using a standard lithium acetate protocol, and transformants were selected for a Leu^+^ prototroph phenotype. At least three independent transformants were analyzed for each plasmid and genotype combination.

### Sorting of mother and daughter cells

Cells were labeled with biotin, grown overnight, labeled mother cells were separated from daughter cells using magnetic anti-biotin microbeads and magnetic columns, and relative replicative age was determined by flow cytometry of cells stained with an Alexa Fluor 488 conjugate of wheat germ agglutinin (WGA, Life Technologies), as previously described [5]. Two exceptions were that each population was split into four five mL cultures, rather than a single 20 mL culture, for overnight growth to reduce the chance that an early Ty1 retromobility event would result in an unusually high retromobility frequency for a population, and cells were never incubated on ice before or after sorting. Mother cell enrichment was estimated by determining the percentage of each eluted mother cell population that exhibited a WGA fluorescence signal higher than 90% of the corresponding daughter cells washed off the column.

### Fractionation and analysis of quiescent cells

Cells were inoculated at a density of 5,000 cells/mL in 20 mL of liquid medium in 125 mL flasks and grown for seven to eight days at 20˚C, at which point quiescent and nonquiescent cells were separated using Percoll density centrifugation, as previously described [19]. Fractionated cells were diluted in five mL of water, centrifuged, washed twice with one mL of water, and suspended in one mL water. Thermotolerance was determined by incubating cells in water at 52˚C for 20 minutes, spreading dilutions of cells on YPD agar to determine colony-forming-units (cfu)/mL, and dividing those values by the cfu/mL of untreated cells to determine the percentage of thermotolerant cells. Cell budding was determined by light microscopy and counting the fraction of cells with buds that were equal to or less than 50% of the size of the mother cell (small-budded cells) or with buds greater than 50% of the size of the mother cell (large-budded cells). Unfractionated cells from *S. paradoxus* strains with different Ty1 copy numbers grown for seven days at 20˚C in YPD were diluted 100-fold into 20 mM EDTA and incubated for five minutes with continuous vortexing to disrupt clumped cells prior to determining the number of budded cells. Relative replicative age was determined as for mother and daughter cell sorting. Plating efficiencies were determined by using light microscopy to count cells, spreading known numbers of cells on solid rich medium, and incubating the cells for four days at 30˚C to allow colonies to form. The number of colonies that formed was divided by the total number of cells spread on the medium to calculate the plating efficiency.

### Ty1 retromobility frequency and rate

The frequency and rate of retromobility of the chromosomal Ty1*his3AI* element in JC3787, JC3212, and their derivatives were measured by selecting for His^+^ prototrophs as described previously [5], since retrotransposition of Ty1*his3AI* leads to removal of an intron in the *HIS3* marker gene and integration of a Ty1*HIS3* element [25]. Cells were inoculated into 1-20 mL of the appropriate medium at an initial density of 5,000 cells/mL and incubated at 20˚C [26] on a culture tube rotator or in a shaking incubator for two (mid-exponential), four (early stationary), or seven to eight days (quiescence). Cells were grown in SC minus leucine + 2% glucose for experiments involving the *PMA1* plasmid and empty vector for 64-66 hours or up to 84 hours for the wild type and *brp1∆* strains, respectively, to achieve similar cell densities. For some experiments, retromobility frequency was measured in cells grown in YPD at 20˚C to mid-exponential phase, and then the cells were pelleted and suspended in YPD or YPD lacking glucose, with 10 or 100 mM calcium chloride, 0.5 M sodium chloride, 10 mM manganese chloride, or 10 mM zinc sulfate. After eight hours at 20˚C, the retromobility frequency was measured again, and this value was divided by the initial frequency value to obtain a relative frequency.

### Western blotting

Protein extracts were prepared by solubilizing suspended cells in an equal volume of 1.4 N NaOH and 5% beta-mercaptoethanol on ice, followed by addition of acetone and trichloroacetic acid to precipitate proteins, as described previously [70]. Proteins were separated on 10% (Gag) or 8% (Gag-GFP) SDS-PAGE gels and transferred to polyvinylidene difluoride (PVDF) membranes. Gag protein was detected using a 1:12,500 dilution of affinity-purified anti-Gag polyclonal antibody [11] (kindly provided by Joan Curcio, Wadsworth Center, Albany, NY) diluted in phosphate-buffered saline (PBS) with 0.05% Tween 20 and 1% nonfat milk, followed by incubation with horseradish peroxidase-conjugated secondary antibody and SuperSignal West Pico chemiluminescent substrate (Thermo Scientific). GFP was detected using a 1:10,000 dilution of an affinity-purified polyclonal GFP antibody from rabbits (Sigma) using the same blocking buffer and subsequent steps as for Gag Westerns. Blots were exposed to film, films were imaged using a Bio-Rad Gel Doc EZ Imager, and signals were quantified using Image Lab 5.2.1 software (Bio-Rad). Blots were stripped in 50 mM Tris pH 7, 2% sodium dodecyl sulfate, and 2.5% beta-mercaptoethanol at 70˚C for 30 minutes, washed in several changes of PBS, and reprobed with a 1:7,500 dilution of a mouse monoclonal anti-beta actin antibody (Abcam) as a loading control. Three to six samples were analyzed for all quantifications.

### Determination of chronological lifespan

Triplicate cultures of each strain were initiated at 5,000 cells/ml in five mL of YPD medium and incubated on a rotator at 20˚C. Populations were sampled initially on day 3 and then every 3-14 days thereafter, depending on the strain and point during lifespan. Trypan blue dye exclusion was used to determine cell viability, as described previously [3]. Trend lines were fit to plots of viability against days to determine the days required to reach 50% viability as a representation of median lifespan.

### Determination of growth rates

Cells were inoculated at 5 x 10^5^ cells/mL into five mL of either YPD or yeast extract-peptone-3% glycerol (YP + Gly) in triplicate and incubated on a rotator at 20˚C. Aliquots of cells were counted on hemacytometers by light microscopy at several time points for 32 hours of growth. Population doubling times in hours were calculated by fitting exponential trend lines to plots of the cell densities over time. The reciprocal of the doubling time was calculated to report growth rates as doublings per hour.

### Statistical analysis

Mean values were compared for significant differences using unpaired two-tailed t-tests assuming equal or unequal variance, based on an f-test calculation for each pair of data sets. Levels of significance and the number of trials for each set of experiments are given in figure legends.
